# Allometric association between physical fitness test results, body size/shape, biological maturity, and time spent playing sports in adolescents

**DOI:** 10.1371/journal.pone.0249626

**Published:** 2021-04-06

**Authors:** Matteo Giuriato, Adam Kawczynski, Dariusz Mroczek, Nicola Lovecchio, Alan Nevill

**Affiliations:** 1 Department of Neurosciences, Biomedicine and Movement Sciences, University of Verona, Verona, Italy; 2 Faculty of Physical Culture, Department of Health and Natural Sciences, Unit of Molecular Biology, Gdansk University of Physical Education and Sport, Gdansk, Poland; 3 Department of Paralympics Sports, University School of Physical Education, Wroclaw, Poland; 4 Laboratory of Adapted Motor Activity (LAMA), Department of Public Health, Experimental Medicine & Forensic Science, University of Pavia, Pavia, Italy; 5 Dep. of Human and Social Science, University of Bergamo, Bergamo, Italy; 6 The Faculty of Education, Health and Wellbeing, University of Wolverhampton, Wolverhampton, United Kingdom; Universidade Federal de Mato Grosso do Sul, BRAZIL

## Abstract

Regular participation in strength and conditioning activities positively correlates with health-related benefits in sports (team and individual). Maturity offset (MO) is a recognized parameter in fitness outcome assessment. The aims of the present study are to analyze cross-sectional allometric development of motor performances in a sample of adolescents and relate scaled motor performance to the estimated amount and type of physical activity and biological maturity status in 771 subjects aged 14–19 years. Three physical fitness components were evaluated using field tests (standing broad jump, sit-ups, shuttle run). Extra hours of sport after school (EHS) and MO were the covariates. The model to predict the physical performance variables was: Y = a · M^k1^ · H^k2^ · WC^k3^ · exp(b · EHS + c · MO) · ε. Results suggest that having controlled for body size and body shape, performing EHS and being an early developer (identified by a positive MO slope parameter) benefits children in physical fitness and motor performance tasks.

## Introduction

Physical activity is positively associated with good health outcomes for children and young people: psychosocial and cognitive development [1], fitness [2], bone/skeletal health [3], and cardiometabolic health [1]. Youth athletes, for example, gain psychological skills essential for participation in individual sports [4]. There is a growing body of evidence for a positive correlation between regular engagement in strength and conditioning activities (adjusted for age) and health-related benefits: increased strength, reduced injury risk, improved metabolic profile of diabetes and its management [3]. It has also been noted that young children with a low level of motor skills are likely to become sedentary adolescents [5,6].

A robust classification of physical activity includes the type of activity, with different training plans for team or individual sports practice. Individual sports athletes train for months to achieve peak fitness and performance in a single event or a series of events [7,8]. Differently, team sports athletes follow in-season and off-season training plans in preparation for the competitive season [8]. A recognized parameter in fitness outcome assessment is maturity offset (MO), defined as the time before or after peak height velocity (PHV) [7], i.e., the period of fastest growth during puberty [7]. Adjusting physical fitness test results to a child’s age at PHV rather than chronological age may be a better approach to determine trends for speed and power during growth [9]. Physiological determinants influence strength outcomes, with the peak of strength corresponding to MO (adolescence) [10]. For example, Mirwald [11] reported a spurt in $\dot{\text{V}}{\text{O}}_{2}\text{max}$ concomitant with PHV (for both sexes). Since higher $\dot{\text{V}}{\text{O}}_{2}\text{max}$ levels during adolescence correspond to a higher value in adulthood, studies involving children and adolescents in youth sport classifications should apply the biological maturity criteria [12].

Moreover, fitness performance is also influenced by an individual’s somatotype [12]. Multilevel analysis of motor performance data considers the effects of developmental changes in young athletes [12,13]. The use of a standardized ratio alone (e.g., $\dot{\text{V}}{\text{O}}_{2}\text{max}$ per kilogram of body mass) can lead to data mismatching and inappropriate conclusions. Armstrong and Welsman [14] suggested that peak VO_2_ increases with gender-specific, age-driven and maturity status-driven concurrent changes in morphological covariates, with the timing of these alterations specific to individuals. Furthermore, based on ratio-scaled peak oxygen uptake, the delivery/utilization of oxygen is facilitated by increased fat-free mass, thus favouring lighter weight and penalizing heavier youths [12,13].

To avert arriving at incorrect conclusions and misinterpretation of data, and additive polynomial model has been developed to analyze and interpret data [14–17] (during childhood/adolescence) and appropriately separate the developmental growth and maturation factors from other components. For a brief history of allometric modeling, see Winter and Nevill [15]. Taking this approach, Silva [16], Lovecchio et al. [17], and Giuriato et al. [18] used multilevel allometry to describe changes in motor performance during childhood in sedentary adults. They found that the optimal body shape associated with physical performance (lower limb explosive strength, trunk strength, endurance performance) is the ectomorph (linear physique): a lean and taller subject.

In the present study, we analyzed the allometric development of motor performance (lower limb explosive strength, trunk strength-endurance, speed-agility) in a sample of adolescents and related the scaled motor performance to the estimated amount and type of physical activity and biological maturity status. We believe that an analysis based on a non-linear approach can account for the exponential growth changes that affect sports performance. In addition, determining the physical activity with the most effective impact on young persons’ sports performance could provide a useful guide for health mentors.

## Materials and methods

### Participants

The study was conducted in January 2019 and involved 771 adolescents (401 boys and 331 girls, age range, 14–19 years) attending a high school in northern Italy. Inclusion criteria were: physical fitness certificate; regular physical education (PE) class attendance; no physical impairments or illnesses or neurological disorders; if history of orthopedic injury, then older than 1 year before inclusion in the study.

The cross-sectional cohort design provides a descriptive measure for boys and girls separately. The Ethics Committee approved the study of the Lombardy Regional School Board (UP 1819–15). Teachers and students received written information about the study purpose and procedure. Informed consent was obtained from participants’ parents or legal guardians. Participants were free to withdraw from the study at any time.

### Procedure

Tests were conducted by a team of master’s degree students in PE during school time for PE [19]. The students had received training to ensure accuracy and repeatability of the test procedures [20] and were supervised by a senior researcher [21]. School PE teachers helped ensure compliance with the study procedures [21]. Anthropometric (body mass and height) measurement and physical fitness assessment (three field tests) were done for 4 weeks, with one round of measurements taken per week to avoid fatigue phenomena [22].

### Anthropometric measures

Anthropometric measurements were taken by trained operators (quality-control coefficient for inter-and intra-observer reliability, 95% confidence interval). Standing height was measured to the nearest 0.5 cm (Seca Stadiometer 208) without shoes, feet together, and head in the Frankfort plane. Body mass was measured to the nearest 0.5 kg (Seca Beam Balance 710), with participants wearing minimal clothing. Waist circumference (WC) was measured over the naked skin with flexible bands (Seca) to the nearest 0.1 cm, half-way between the lower rib and the top of the iliac crest at the end of gentle expiration, according to Fredrik’s guidelines [22,23].

### Measures of physical fitness

Three physical fitness components were evaluated using field tests from the Eurofit test battery [24], as administered in a similar previous study [25,26]. All three tests are reliable, valid instruments to measure physical fitness [19,26] and are considered independent of each other. A brief explanation is given below.

*Standing Broad Jump* (SBJ, lower limb explosive strength, systematic error near zero [27]; cm): from a standing position immediately behind a line with feet approximately shoulder-width apart, the participants jumped as far as possible with feet together. The rearmost foot is taken as the measure (centimetres). Swinging the upper limbs during the jump is permitted.

*Sit-Ups* (SUP, trunk strength-endurance, ICC ranged from 0.85 to 0.98 [28]; n): The efficiency of abdominal musculature is measured by the maximum number of sit-ups (crunch) achieved within 30 s. The starting arrangement involves the subject taking a lying position, fingers interlocked behind the nape, knees bent at a 90° angle, and heels/feet flat on the floor. The subject rises from the lying to the sitting position with the elbows extended so that they touched the knees. The total number of sit-ups performed correctly within 30 s is recorded.

*Shuttle Run Test 10 x 5 m* (SHR, speed-agility, 0.8 to 4.0% of CV [29]; s): Two parallel lines (2 meters long) are drawn on the floor 5 m apart. The subject runs as fast as possible from the starting line to the other and returns to the starting line, crossing each with both feet each time for a total of 10 times. The stopwatch is stopped when the subject crosses the finishing line with one foot. The test is recorded to the nearest 0.1 s.

### Sports participation

#### Extra hour sport (EHS) after school

Data on physical activity and sports participation were collected via a self-reported questionnaire [30] administered to each participant 1 week before the physical fitness assessment. As described in Telama et al. [30], the questionnaire contained items on the weekly frequency of sports after school hours and whether in team or individual sports.

#### Open and closed skills (OCS)

Sports can be divided into two categories according to the effects of the environment on motor skills: open and closed sports [28]. Open sports are characterized by skills performed in a dynamic and changing environment (e.g., soccer or basketball), while closed sports take place in a predictable and static environment (e.g., sprint or gymnastics) [28].

#### Individual and team sport (ITS)

Sports can also be divided into two categories according to competitions: individual and team sports. Individual sports are characterized by individual performance against another single participant, while team sports involve two or more members who compete against the opposite team [29].

### Data analysis

Continuing debate surrounds the interpretation of the influence of body size, composition, and shape on physical fitness testing of children and adolescents of different socioeconomic status [31] and cultural background [6]. The allometric approach, adopted in the present study, is currently viewed as a suitable model to help solve this issue, given the model’s sound theoretical basis, biological underpinnings, and its elegant and versatile statistical methodology [12,13]. Demographic data, anthropometric measures, information about sports participation, and physical fitness test results were stratified for all participants. Biological maturation was estimated using the somatic maturation method described by Moore [32], which estimates the MO from stature and chronological age according to an algorithm and provides a number of years to PHV. The participants were classified as “late”, “early” or “on time” developer according to the standard deviation method derived from the current sample [8].

### Statistical methods

Data analysis was performed using SPSS Statistics (IBM Corp. Released 2017. IBM SPSS Statistics for Windows, Version 25.0. Armonk, NY: IBM Corp). The multiplicative model (Eq 1) with allometric body-size components of body mass (M), height (H), and waist circumference (WC) was used to identify the most appropriate body size and shape (somatotype) characteristics. The model was associated with MO, EHS, and categorical differences (sex, age, team vs individual and open vs closed skills) as covariates. The model, here applied to the three tests (SBJ, SUP and SHR), is an extension of the one used to predict physical performance variables in Greek children [33]:

$$\text{Y}=\text{a}·{\text{Mk}}_{1}·{\text{Hk}}_{2}·{\text{WCk}}_{3}·\text{exp}(\text{b}·\text{EHS}+\text{c}·\text{MO})·\varepsilon$$ (1)

**Fig 1:**
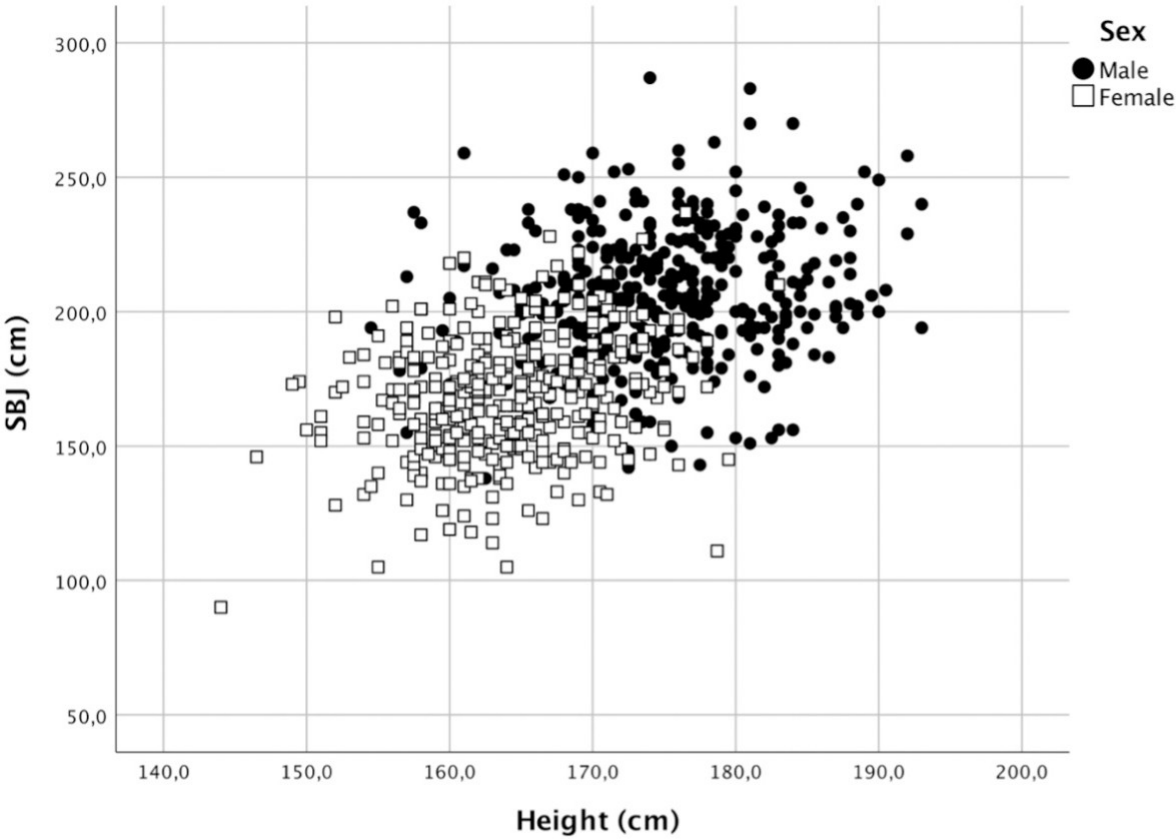
Association between height (cm) and SBJ (cm) for boys and girls.

The advantage of the model is that it has proportional body-size components and a multiplicative error that assumes that ε increases proportionally with the physical performance variable Y (e.g., see Fig 1).

The model (Eq 1) can be linearized with a log transformation (Ln = log_e_). Linear regression analysis or analysis of covariance (ANCOVA) of Ln(Y) can then be used to estimate the unknown parameters of the log-transformed model:

Ln(Y)=Ln(a)+k1·Ln(M)+k2·Ln(H)+k3·Ln(WC)+b·EHS+c·MO+Ln(ε) (2)

Other categorical or group differences within the sample (sex, OCS or ITS) can be explored by allowing the constant intercept parameter Ln(a) to vary for each group by introducing them as fixed factors (plus possible interactions) within an ANCOVA (note that the log-transformed data for the SBJ, SUP, and SHR were incorporated as the Ln(Y) dependent variables and the terms Ln(M), Ln(H), Ln(WC), EHS, and MO in Eq (2) were adopted as the covariates). The significance level was set at *P*<0.05.

## Results

Table 1 presents the mean (± standard deviation, SD) physical fitness test results by sex, OCS, and ITS.

**Table 1:** Physical fitness outcomes (Mean ± SD) by sex, type of sport, and type of activity.

Performance	Sex		Type of sport		Type of activity	
	Boys (n = 401)	Girls (n = 370)	Open Skill (n = 346)	Closed Skill (n = 257)	Individual (n = 317)	Team (n = 286)
SBJ (cm)	205.80	168.27	198.32	182.71	183.62	199.91
	24.51	22.71	25.42	31.02	30.51	24.77
SUP (n)	23.48	20.35	23.10	21.83	21.90	23.23
	4.04	4.00	3.94	4.50	4.56	3.75
SHR (s)	19.34	21.34	19.59	20.66	20.67	19.40
	1.60	1.71	1.72	1.84	1.84	1.61

### Standing broad jump test

Table 2 presents the estimated parameters from the multiplicative model relating the SBJ distance to the body-size components in Eq 1, incorporating EHS and MO.

**Table 2:** SBJ performance: Parameter of Eq 1 based on females.

Parameter	B	SE	t	p-value	95% Confidence Interval	
					Lower Bound	Upper Bound
Intercept Ln(a)	2.697	.793	3.400	.001	1.140	4.255
Male ΔLn(a)	.135	.021	6.529	.001	.095	.176
Ln(M) (k_1_)	-.125	.057	-2.198	.028	-.236	-.013
Ln(H) (k_2_)	.700	.150	4.664	< .001	.405	.995
Ln(WC) (k_3_)	-.166	.084	-1.978	.048	-.330	-.001
EHS (b)	.010	.002	5.865	< .001	.006	.013
MO (c)	.019	.006	3.230	.001	.007	.030

Parameters for girls are not given because considered as offset.

The multiplicative model (Eq 1) relating the SBJ distance to the body-size components was:

$$\text{SBJ}\;\text{distance}\;(\text{cm})=\text{a}·{\text{M}}^{-0.125}·{\text{H}}^{0.700}·{\text{WC}}^{-0.166}$$

With positive height (H) and negative mass (M), and waist (WC) exponents, the model suggests that being taller but lighter (less body mass) with a smaller waist circumference is the optimal body shape associated with SBJ.

**Fig 2:**
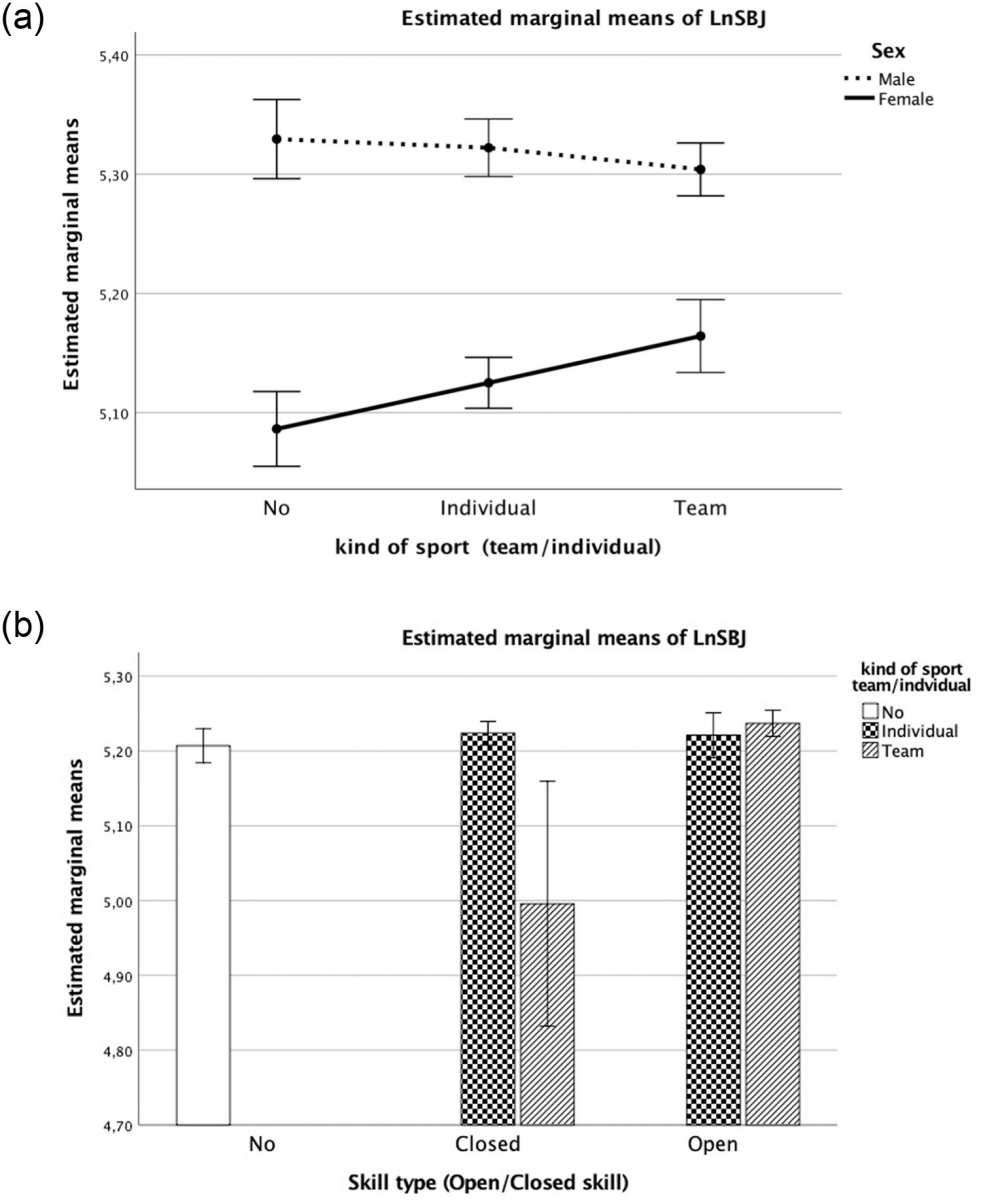
a. Estimated marginal means of SBJ test by type of sport (team/individual) and sex (boys/girls). Covariates appearing in the model are evaluated as: LnWC = 4.2640, LnHT = 5.1327, LnMass = 4.1025, Extra school hours of sports = 4.311, Maturity offset = 2.9637, MO2 = 10.0568. b. Estimated marginal means of SBJ by type of skill (open/closed) and sport (team/individual). Covariates appearing in the model are evaluated as: LnWC = 4.2640, LnHT = 5.1327, LnMass = 4.1025, Extra school hours of sports = 4.311, Maturity offset = 2.9637, MO2 = 10.0568.

Fitting Model (Eq 1) revealed significant differences in the constant “a” parameter due to sex (P <0.001), together with two interactions between the categorical variables “sex” and “type of sport” (Team vs Individual) (P <0.001; Fig 2a) and “Open vs Closed Skill” and “type of sport” (Team vs Individual) (P <0.05).

A significant positive contribution to predict log-transformed SBJ distance was noted for extra hours of sports (EHS) and maturity offset (MO) (both P <0.001).

### Sit-up test

Table 3 presents the estimated allometric parameters from the model relating the number of SUP to the body-size components in Eq 1, incorporating EHS and MO.

**Table 3:** SUP performance: Parameter of Eq 1 based on females.

Parameters	B	SE	t	P-value	95% Confidence Interval	
					Lower Bound	Upper Bound
Intercept Ln (a)	3.696	.309	11.958	< .001	3.090	4.303
Male ΔLn (a)	.142	.029	4.912	< .001	.086	.199
Ln (WC) (k_3_)	-.161	.075	-2.149	.032	-.308	-.014
EHS (b)	.015	.002	6.070	< .001	.010	.019
MO (c)	-.030	.007	-4.338	< .001	-.043	-.016

Parameters for girls are not given because considered as offset.

The multiplicative model relating the number of sit-ups performed in 30 s (SUP) to the body-size components using Eq 1 identified waist circumference as the only significant body-size component k3 = -0.161 (SEE = 0.075).

**Fig 3:**
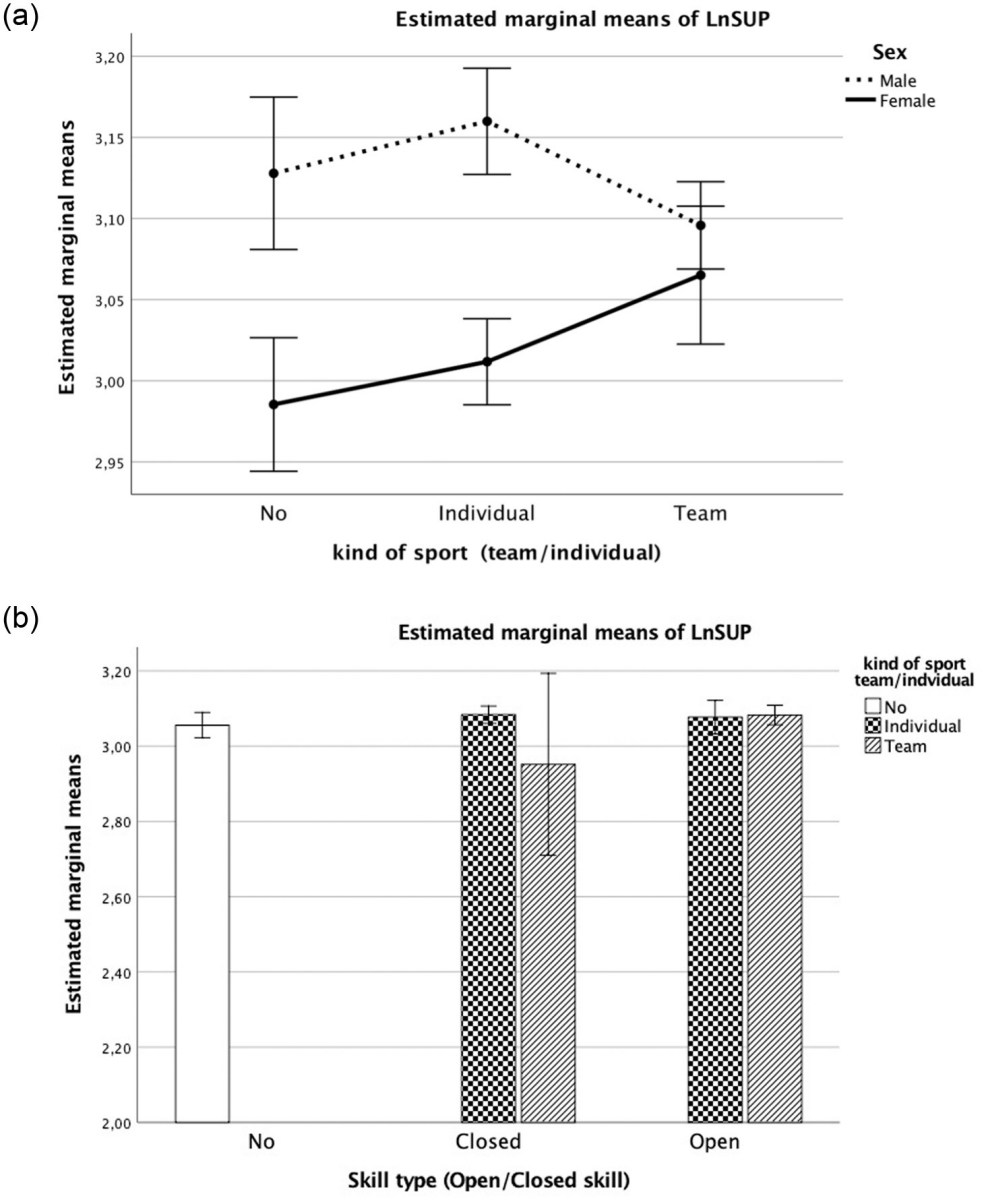
a. Estimated marginal means of SUP test by type of sport (team/individual) and sex (boys/girls). Covariates appearing in the model are evaluated as: LnWC = 4.2640, Extra school hours of sports = 4.311, Maturity offset = 2.9637. b. Estimated marginal means of SUP test by type of skill (open/closed) and sport (team/individual). Covariates appearing in the model are evaluated as: LnWC = 4.2640, Extra school hours of sports = 4.311, Maturity offset = 2.9637.

The model (Eq 1) fitted to the log-transformed SUP also revealed that the constant “a” parameter varied by sex (P <0.001), together with an interaction between the categorical variables “sex” and “kind of sport” (Individual vs. Team) (P <0.001; Fig 3).

A significant contribution to predict log-transformed SUP distance was noted for extra hours of sports (EHS) and maturity offset (MO) (both P <0.001). As expected, EHS was positively related, whereas MO was negatively related to the number of sit-ups in 30 s.

### Shuttle Run Test 10x 5m (SHR)

Table 4 presents the estimated allometric parameters from the model relating shuttle-run-test performance (mean speed, in m.s-1) and body-size components in Eq 1, incorporating EHS and MO.

**Table 4:** SHR performance: Parameter of Eq 1 based on females.

Parameter	B	SE	t	P-value	95% Confidence Interval	
					Lower Bound	Upper Bound
Intercept Ln (a)	-1.247	.084	-13.7	< .001	-1.412	-1.083
Male ΔLn (a)	.100	.008	-12.6	< .001	-.116	-.085
Ln (M) (k_1_)	-.059	.021	-2.8	.006	-.100	-.017
EHS (b)	.006	.001	6.0	< .001	.004	.008
MO (c)	.009	.003	2.8	.050	.003	.016

Parameters for girls are not given because considered as offset.

**Fig 4:**
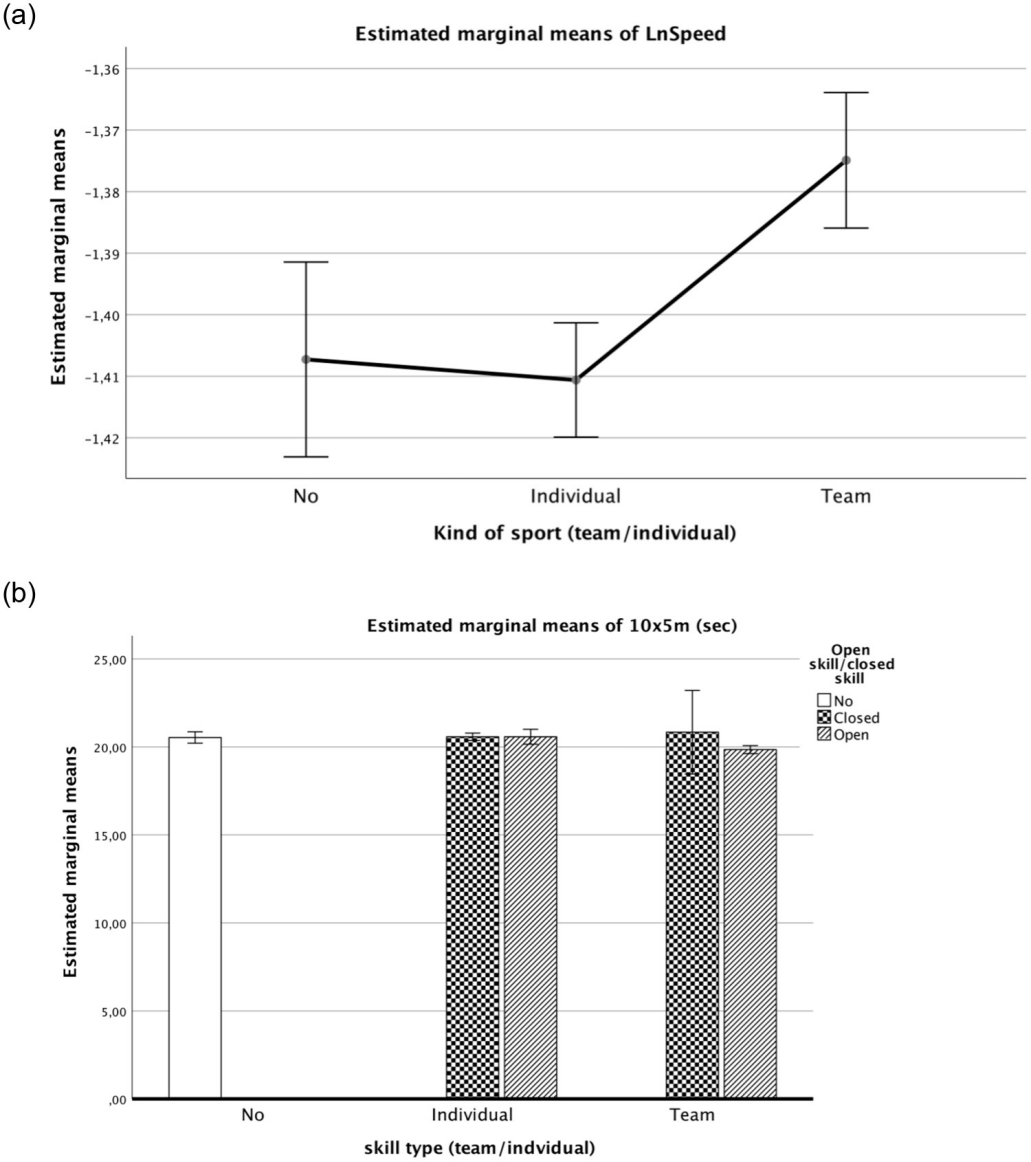
a. Estimated marginal means of SHR test by type of sport (team/individual). Covariates appearing in the model are evaluated as: LnMass = 4.1025, Extra school hours of sports = 4.311, Maturity offset = 2.9637. b. Estimated marginal means of SHR test by type of skill (open/closed) and sport (team/individual). Covariates appearing in the model are evaluated as: LnMass = 4.1025, Extra school hours of sports = 4.311, Maturity offset = 2.9637.

The model relating the shuttle-run-test performance (using mean speed, in m.s-1) to the body-size components using Eq 1 identified body mass as the only significant body-size component k3 = -0.059 (SEE = 0.021). The model (Eq 1) fitted to the Ln-transformed SHR also revealed that the constant “a” parameter varied by “sex” (P <0.001) and “type of sport” (individual vs team) (P <0.001; Fig 4).

A significant contribution to predict log-transformed SHR speed was noted for extra hours of sports (EHS) and maturity offset (MO) (both P <0.001). As expected, EHS and MO were positively related to the shuttle run speed.

## Discussion

With this study, we investigated the relationships between biological maturation/body shape characteristics (height, mass, WC) and physical fitness using field tests (Eurofit battery; SBJ, SUP, SHR 10x5m) in a cohort of adolescents [24]. The allometric modeling approach to determine the influence of sports involvement outside school hours takes into account different types of skill sets (open, closed) and sports (team or individual) [12–14]. Our findings suggest that fat body mass negatively affects physical fitness in adolescents. The allometric model showed that the earlier developers were better in speed-agility (SHR 10x5m) and explosive strength (SBJ) and that the late developers outperformed the others only on the SUP trials. This difference could be due to less body mass and delay in maturation: taller and more mature children have longer legs [33] and more potential strength to perform sprint and jumps at the core of the three trials. Furthermore, somatotype derives from allometric modeling through the exponent in Eq (1), as Winter and Nevill reported [15]. Previous studies suggested that linear/long-limbed subjects (ectomorphic) are more efficient in open skill sports [34]. That require rapid changes in direction, jumps, decelerations, and fast reactions [7]. The somatotype results from the interpretation of exponents in Eq 1 and Table 2, specifically from the B parameter of LnM (.-125) and LnH (.700), are shared by Winter and Nevill [15].

The ectomorphic subjects performed better on the SBJ (Eq 1) and the SHR test (Eq 2). These observations are in line with previous data [12–18,35] for biological maturity, whereas performance on the SBJ in subjects at an early stage of maturation revealed an exponent of 0.7 (Eq 1).

Nevill et al. [35] suggested talent identification procedures to recognize more ectomorphic players at an early age. In soccer (open skills sport), for example, explosive strength and speed-agility are essential. We observed that the smaller subjects (less body mass due to early maturation) performed better on the SBJ and the SHR test. The negative MO suggests that late mature youths may do better on the SUP test (Table 3). We may speculate that while late developers have longer legs, more importantly, they have a relatively short upper torso (less upper body mass to raise doing sit-ups). Furthermore, sports coaches need to consider the demands of the modern understanding of sports; fast actions rely on speed and agility [36], where team sport-specific movements are typically observed in the players with a small body size [34,35].

In the comparison of the differences between males and females, the boys appear more ectomorphic [16]. The anthropometric data show that boys are taller than girls and have more fat-free mass, which is advantageous for sports performance [34]. Similar sex-related differences were found for sedentary individuals’ performance on the SUP test; boys outperformed girls, as indicated by WC (less body mass is a good predictor). The boys outperformed the girls also on the speed-agility test; biological factors (e.g., testosterone production) can also affect the high-speed change of direction (an important asset in open sports) [6,7].

Extra hours of sports were the first predictor of performance on the three tests (SBJ, SUP, SHR 10x5m) (Figs 2–4, respectively). The covariates showed that EHS are fundamental for the development of adequate strength in both team and individual sports. Sports coaches should focus on strength conditioning programs starting already in preadolescence [15]. Further studies on EHS and strength training in youths may provide additional evidence for fitness improvement (i.e., progression in skills and strength conditioning) and associated health-related benefits. Moreover, extending research to youth athletes could advance our understanding of the magnitude of EHS as the first predictor. Our findings for EHS as the first predictor of performance based on strength suggest that the time spent playing sport can be usefully employed to enhance performance (e.g., lower limb explosive strength, speed agility, trunk strength endurance). An ectomorphic body shape is the best physique for better performance [16,17,35,36]. While there was a significant contribution of MO to explosive strength and speed (SBJ, SHR) performance (see parameter B in Tables 2 and 4), it did not affect strength-endurance (SUP) (see parameter B in Table 3). A positive score of greater MO (earlier maturation) on the SBJ and the SHR test (Tables 2 and 4) improved the outcome on these fitness tasks.

## Conclusions

One limitation of the present study is that the self-report questionnaire could have been more robust in design (like the International Physical Activity Questionnaire (IPAQ) developed as an instrument for cross-national monitoring of physical activity and inactivity). Moreover, physical fitness assessment should include an aerobic test. However, due to the large amount of data and the school setting of the study, these choices were made to promote student compliance.

This study provides information about strength status in adolescents: children with an ectomorphic body shape, due to their taller physique and reduced body mass, performed better on the speed-agility and the explosive strength tests, as reported previously [36]. A linear body shape and engagement in EHS could provide useful clues for talent identification. Finally, our findings suggest that strength tests may be helpful to identify potential motor performance talents in earlier developers.
